# LIMD2 Regulates Key Steps of Metastasis Cascade in Papillary Thyroid Cancer Cells via MAPK Crosstalk

**DOI:** 10.3390/cells9112522

**Published:** 2020-11-23

**Authors:** Rodrigo Pinheiro Araldi, Thatiana Correa de Melo, Débora Levy, Dener Madeiro de Souza, Beatriz Maurício, Gabriel Avelar Colozza-Gama, Sergio Paulo Bydlowski, Hongzhuang Peng, Frank J. Rauscher, Janete Maria Cerutti

**Affiliations:** 1 Genetic Bases of Thyroid Tumors Laboratory, Division of Genetics, Department of Morphology and Genetics, Universidade Federal de São Paulo/EPM, São Paulo, SP 04039-032, Brazil; rodrigo.pinheiro.araldi@gmail.com (R.P.A.); avelarbio46@gmail.com (G.A.C.-G.); 2 Programa de Pós-graduação em Biociências, Universidade Federal da Integração Latino-Americana (UNILA), Foz do Iguaçu, PR 85866-000, Brazil; thatiana.c.melo@gmail.com; 3 Laboratory of Histocompatibility and Cellular Immunity, Faculdade de Medicina, Universidade de São Paulo (USP), São Paulo, SP 05404-000, Brazil; d.levy@hc.fm.usp.br (D.L.); spbydlow@usp.br (S.P.B.); 4 Genetics Laboratory, Instituto Butantan, São Paulo, SP 05503-900, Brazil; dener.souza@butantan.gov.br; 5 Laboratory of Cell Biology, Instituto Butantan, São Paulo, SP 05503-900, Brazil; beatriz.mauricio@butantan.gov.br; 6 The Wistar Institute, Philadelphia, PA 19104, USA; hongzhuangpeng851@gmail.com (H.P.); fjr88911@icloud.com (F.J.R.III)

**Keywords:** papillary thyroid carcinoma, LIMD2, BRAF V600E, CRISPR/Cas9, epithelial-to-mesenchymal transition

## Abstract

Although papillary thyroid carcinoma (PTC) has a good prognosis, 20–90% of patients show metastasis to regional lymph nodes and 10–15% of patients show metastasis to distant sites. Metastatic disease represents the main clinical challenge that impacts survival rate. We previously showed that LIMD2 was a novel metastasis-associated gene. In this study, to interrogate the role of LIMD2 in cancer invasion and metastasis, we used CRISPR-mediated knockout (KO) of LIMD2 in PTC cells (BCPAP and TPC1). Western blot and high-content screening (HCS) analysis confirmed functional KO of LIMD2. LIMD2 KO reduced in vitro invasion and migration. Ultrastructural analyses showed that cell polarity and mitochondria function and morphology were restored in LIMD2 KO cells. To unveil the signals supervising these phenotypic changes, we employed phospho-protein array. Several members of the MAPK superfamily showed robust reduction in phosphorylation. A Venn diagram displayed the overlap of kinases with reduced phosphorylation in both cell lines and showed that they were able to initiate or sustain the epithelial-mesenchymal transition (EMT) and DNA damage checkpoint. Flow cytometry and HCS validation analyses further corroborated the phospho-protein array data. Collectively, our findings show that LIMD2 enhances phosphorylation of kinases associated with EMT and invasion. Through cooperation with different kinases, it contributes to the increased genomic instability that ultimately promotes PTC progression.

## 1. Introduction

Although most patients with papillary thyroid carcinoma (PTC) have a favorable prognosis, 30–90% of patients exhibit clinical or occult cervical lymph nodes [1,2]. In addition, 1.0–13% of patients present distant metastasis at time of diagnosis, which negatively impacts the survival rate [3].

Moreover, the American Thyroid Association (ATA) thyroid cancer guidelines proposed a dynamic risk stratification scheme that included additional prognostic variables such as the extent of lymph node involvement (i.e., number and size) and mutational status of the primary tumor to determine the risk of disease recurrence or persistence [4,5]. Although metastasis is the main cause of recurrence, failure of cancer therapy, and mortality, it remains poorly understood.

To learn how PTC spreads to the lymph nodes, we performed gene expression profiling on matched normal thyroid, primary PTC, and its lymph node metastasis to identify genes associated with the metastatic process. Several transcripts were highly expressed in lymph node metastasis, comprising *LIMD2* [6]. The expression of *LIMD2* was subsequently confirmed in over 80% of the metastatic PTC and in nearly 95% of matched lymph node metastases. Remarkably, its expression was higher in PTC samples and papillary thyroid cell lines harboring the BRAF V600E mutation than its expression in PTC harboring RET/PTC fusion. Using the Cancer Genome Atlas (TCGA) dataset, we provided further evidence that *LIMD2* expression was higher in BRAF-like than in RAS-like PTC [7].

LIMD2 overexpression has been correlated with a higher degree of invasiveness in breast and melanoma cancer cell lines. The authors showed that LIMD2 controlled the acquisition of multiple hallmarks of tumor progression as anchorage-independent growth and increased migration of different cancer types and cell lines [8]. However, the signaling pathway through which LIMD2 promotes morphological changes to trigger the metastatic behavior still remains unknown.

LIMD2, is a LIM domain protein, which is defined by the presence of one segment containing two adjacent cysteine-histidine-rich zinc fingers separated by a hydrophobic linker that functions as a protein-binding interface, as previously revisited [9]. With the potential ability to bind a wide variety of partners, the LIM domain proteins have been enrolled in different cellular processes including control of gene transcription, cytoskeleton organization to regulate cell growth, motility and division, cell lineage specification, and organ development [10].

The molecular function of the LIM domain is dependent upon the binding of target proteins and because understanding this process would help to identify targets for molecular therapies, in this study, we used CRISPR/Cas9-mediated knockout (KO) of LIMD2 in two PTC cell lines to explore the phosphorylation state of multiple kinases associated with the three major families of MAPK that are associated with cell proliferation and differentiation, cell survival, and cell migration and invasion. We additionally explored the effect of LIMD2 KO on cell ultrastructure, invasion, as well as the expression of key proteins associated with EMT and genomic instability.

## 2. Materials and Methods

### 2.1. Ethical Approval

The study was approved by the Research Ethics Committees of the Escola Paulista de Medicina (EPM), Universidade Federal de São Paulo (UNIFESP, CEUA 9220210917).

### 2.2. Cell Line and Culture

Human thyroid carcinoma cell lines (BCPAP, TPC1, FTC133, FTC236, FTC238, WRO, and TT) were maintained at 37 °C, in a 5% CO_2_ humidified atmosphere. The original histological subtype, the medium in which they were maintained, the source, and the mutational profiling of each cell line are listed in Table 1. Short tandem repeat (STR) profiling was performed for the cell line authentication and to check for cross-contamination.

### 2.3. Western Blotting

The cell homogenates prepared from cell lines were incubated in RIPA buffer supplemented with phosphatase inhibitor cocktails (5 µg/mL aprotinin, 1 µg/mL leuptinin, 1 mM PMSF, and 10.28 mM sodium orthovanadate) and centrifuged at 14,000 rpm for 10 min at 4 °C. The protein concentration was determined using the Pierce BCA Protein Assay Kit (Thermo Fisher Scientific, Carlsbad, CA, USA), according to the manufacturer’s instructions. Then, 50 µg of reduced protein was loaded into 12% gel, transferred to a nitrocellulose membrane (BioRad, Hercules, FL, USA), and blocked for 1 h in TBS-T with 0.5% nonfat dry milk. The membranes were incubated with primary antibodies overnight at 4 °C, washed, and incubated with HRP-conjugated anti-rabbit IgG antibody. The antibodies specifications and working dilutions are detailed in Table 2. Signals were visualized using an Immobilon Western System (Millipore, Burlington, VT, USA) and images were captured using an ImageQuant LAS 4000 (GE Healthcare Bio-Sciences, Uppsala, Sweden). Expression levels of LIMD2 were quantified by pixel densitometry using ImageJ software [12].

### 2.4. RNA Extraction, cDNA Synthesis, and Quantitative RT-PCR

Total RNA was isolate from the thyroid cell lines using TRIzol reagent (Thermo Fisher Scientific), following the manufacturer’s instructions. Total RNA (1 μg) was treated with DNAse and reverse transcribed into cDNA with 50 μM oligo(dT)20 using a Superscript IV transcriptase kit (Thermo Fisher Scientific). About 2 μL of cDNA was used in a 12 μL PCR reaction containing 1X SYBR Green PCR Master Mix (Applied Biosystems, Carlsbad, CA, USA) and 10 pmol of each specific primer for *LIMD2* gene. As reference gene, we used ribosomal protein S8 (*RPS8*). The quantitative PCR reaction was performed in triplicate, and the threshold cycle was obtained using QuantStudioTM 12K Flex Software v1.2.2 (Applied Biosystems). The relative expression levels were calculated based on Delta-Delta-CT (ddCT), as previously reported [6].

### 2.5. LIMD2 Copy Number Analysis by qPCR

The copy number state of *LIMD2* was determined using quantitative PCR (qPCR). DNA was isolated from cell lines, as previously described [13]. PCR was performed in a 12 μL PCR reaction containing 10 ng of DNA, 0.2 μM of each specific primer, and 1X SYBR Green PCR Master Mix (Applied Biosystems Foster City, CA, USA). Primers of LIMD2 or endogenous reference gene (*ACTB*) were available on request. The PCR reactions were run on the QuantStudio 12K Flex system. The relative copy number state was calculated using 2^(−ΔΔCt)^. Copy numbers below 0.5 were defined as lost/deleted and above 1.5 were defined as gained/amplified. DNA isolated from peripheral blood mononuclear cells (PBMC) from three adult women was used as the control [14].

### 2.6. Generation of Stable LIMD2 Knockout (KO) Papillary Thyroid Cell Lines with CRISPR/Cas9 System

Four CRISPR RNA (crRNA) sequences, with 20 ribonucleotides each, were designed using the GeneArt CRISPR Search and Design Tool (Thermo Fischer Scientific, Waltham, MA, USA, https://apps.thermofisher.com/apps/crispr/index.html). The crRNA sequences, which provide the targeting function of the guide RNA (gRNA), are described in Table 3. The guide RNA (gRNA) sequence was prepared as follows: 100 pmol/µL of each crRNA were independently annealed with 100 pmol/µL of trans-activating CRISPR RNA (tracrRNA) using a 4-step program, i.e., 95 °C for 5 min; 95 °C to 78 °C with −2 °C/s; 10 min at 78 °C; and 78 °C to 25 °C with −0.1 °C/s.

The Cas9/gRNA ribonucleoprotein complex was performed using 25 µL of Opti-MEM I medium (Thermo Fisher Scientific), 500 ng of gRNA duplex, 500 ng of GeneArt Platinum Cas9 Nuclease, and 2.5 µL of Cas9 Reagent Plus (Thermo Fisher Scientific) and incubated at room temperature for 5 min. Next, 1.5 µL the lipofectamine CRISPRMAX transfection reagent was diluted in 25 µL of Opti-MEM I medium and incubated at room temperature for 5 min. The Cas9/gRNA complex was coupled with CRISPRMAX transfection reagent, incubated at room temperature for 15 min, and subsequently transfected into 0.4 × 10^5^ cells seeded in a 24-well plate in RPMI 1640 medium.

After 48 h of lipofection, the pool of transfected cells was harvested and counted using the Countess Automated Cell Counter (Thermo Fisher Scientific). To isolate the monoclonal cell line from a CRISPR/Cas9 engineered pooled population, we used the limiting dilution method. Cells were diluted as low as 0.5 cells per aliquot (8 cells/mL). Accordingly, 100 μL of this cell suspension was transferred into each well of a 96-well plate (Corning, New York, NY, USA). Seeding at an average of 0.5 cells/well guaranteed that at least some wells had a single cell. Plates were analyzed in an inverted microscope. The wells with only one cell were further expanded. For each expanded clone, about 20,000 cells were analyzed for the editing efficiency by TIDE, as described in Section 2.7 and Section 2.8.

### 2.7. Analysis of the KO of LIMD2 in Thyroid Cells by PCR Sequencing

Following 48 h post transfection, the DNA was isolated using the phenol-chloroform method, quantified using NanoDrop 2000c (Thermo Fisher Scientific), and amplified by PCR. The PCR product (556 bp) was purified using the QIAquick Gel Extraction kit (Qiagen, Hilden, Germany), sequenced using the BigDye Terminator (Life Technologies, Carlsbad, CA, USA), and analyzed using the ABI 3100 Genetic Analyzer (Applied Biosystems), as previously reported [7].

### 2.8. Tracking of Indels by Decomposition (TIDE) Analysis

Editing efficiency of each gRNA was determined using tracking of indels by decomposition (TIDE) sequencing, using the TIDE Web tool (Netherlands Cancer Institute, Amsterdam, The Netherlands, available from http://shinyapps.datacurators.nl/tide/).

### 2.9. Isolation and Expansion of CRISPR/Cas9 Edited Single Clones Derived from BCPAP and TPC1 Cells

The pool of edited cells that exhibited the highest editing efficiency was used for clonal selection using the limiting dilution method. Then, the clones were subcultured, expanded, and subjected to DNA extraction using the phenol-chloroform method, as previously described [13]. About 100 ng of DNA from each clone was used for PCR amplification and sequenced using the BigDye Terminator (Life Technologies) and analyzed using the ABI 3100 Genetic Analyzer (Applied Biosystems), as previously reported [7]. The DNA sequences obtained from each clone were aligned with the reference sequence of gene *LIMD2* (GenBank ID 80774) using the AliView v. 1.24 software (Uppsala University, Uppsala, Sweden) [15].

### 2.10. Analysis of the LIMD2 Editing Efficiencies Using Western Blot, Flow Cytometry, and High-Content Screening

For flow cytometry, cells (10^6^) were fixed with 1% formaldehyde at 4 °C for 2 h, washed in PBS, and centrifuged at 300× *g* for 5 min at 4 °C. The pellet of cells was incubated with blocking solution (5% BSA) for 40 min at 4 °C, permeabilized with 0.2% Triton X-100 in PBS for 15 min at room temperature, and then incubated overnight at 4 °C with primary antibody. The cells were incubated for 2 h, at 4 °C, with FITC-conjugated goat anti-rabbit IgG and analyzed using an Accuri C6 flow cytometer (BD Bioscience, San Jose, CA, USA) (Table 2). The data were analyzed using the FlowJo software (Tree Star Inc., Palo Alto, CA, USA). Statistical analysis was performed based on the mean of fluorescence intensity (MFI).

For high-content screening (HCS), cells (0.4 × 10^4^) were seeded into a 96-well TC-treated microplates (Corning Costar Corp., Corning, NY, USA). After 24 h, cells were washed with PBS, fixed in 4% formaldehyde, and blocked in 5% BSA for 40 min. The fixed cells were incubated with permeabilization solution (0.01%Triton X-100) for 15 min at 4 °C and labeled with primary antibodies overnight at 4 °C. Then, cells were incubated with FITC-conjugated goat anti-rabbit IgG, washed with PBS, and the nuclei stained by Hoechst 33342 (Thermo Fisher Scientific). In order to analyze the autofluorescence, unstained cells were incubated with fluorochrome-labeled secondary antibody FITC-conjugated goat anti-rabbit IgG, (Table 2). The analyses were performed in duplicate, using the ImageXpress MicroXLS Widefield High-Content Analysis System (Molecular Device, San Jose, CA, USA).

### 2.11. Off-Target Analysis

Possible off-target sites for *LIMD2* gRNA2 were predicted by GeneArt CRISPR Search and Tool. Allowing a maximum of three bases of mismatches, five putative off-target sites were found (Table 4). Two off-target sites (*COLS19A* and *PPAR4*) were PCR amplified from the DNA of gRNA2-transfected clones and analyzed by sequencing.

### 2.12. Ultrastructure of Parental and Edited LIMD2 Cells via High Resolution Imaging Analysis

Morphological and ultrastructural analyzes were performed using the transmission electron microscopy (TEM) and the scanning electron microscopy (SEM). Cells were fixed overnight in 1% glutaraldehyde in 0.1 M PBS and centrifuged at 300× *g* for 10 min. The pellet was washed three times with 0.2 M sucrose in 0.1 M PBS for 15 min, and then incubated for 1 h with OsO_4_ in 0.1 M PB. Then, cells were dehydrated in a graded alcohol series and treated with 100% propylene oxide for 15 min. For TEM, cells were embedded in 1:1 EMBed 812-propylene oxide for 2 h at room temperature and transferred to 2:1 EMBed 812-propylene oxide overnight in a desiccator with the top off. Samples were embedded in beam capsules and allowed to polymerize at 60 °C for 48 h. The embedded cells were sectioned (0.5 µm) and stained with uranyl acetate for 15 min. Cells were analyzed in TEM microscopy Leo 906E (Carl Zeiss, Oberkochen, Germany). For SEM analysis, fixated adherent cells were metalized and analyzed in a QUANTA 250 microscopy (FEI Company, Hillsboro, OR, USA).

### 2.13. Monitoring Cell Invasion

To evaluate the effects of *LIMD2* KO on cell invasiveness, parental and edited cells were analyzed using Transwell assay. Cells were seeded in 24-well permeable inserts with 8.0 µm pore size (Corning Costar Corp.) coated with 100 µL of Matrigel coating solution (Sigma Aldrich, Sannois, France, cat. # E1270) and incubated at 37 °C overnight with serum-free medium. Complete medium was added in the lower chamber. Following incubation for 24 h, cells on the upper side were gently removed by scraping with a cotton swab. The cells on the lower surface of the membrane were fixed in 100% methanol and stained with 100% Giemsa. Cells in three different fields were counted under the microscope at 40× magnifications. As controls, a similar number of cells was seeded in 24-well uncoated permeable support.

### 2.14. Proteome Profiler Antibody Array

For the parallel determination of the relative phosphorylation of 65 multiple kinases associated with the three major families of MAPK, cells lysates were incubated with proteome profiler Human Phospho-MAPK array kit (ARY002B) and the proteome profiler Human Phospho-Kinase Array Kit (ARY003B) (R&D Systems, Minneapolis, MN, Canada), as previously described [16].

Results were analyzed through fold change-based histogram in log scale, both obtained using R-based software. In order to compare the proteome profile of the most dephosphorylated proteins between the different cell lines (BCPAP and TPC1), a Venn Diagram was used, using the web tool Draw Venn Diagram (http://bioinformatics.psb.ugent.be/webtools/Venn).

### 2.15. Statistical Analysis

Statistical analyses were performed using the parametric analysis of variance (ANOVA), followed by the Dun’s post hoc test (both with 5% of significance levels based on the mean of fluorescence intensity (MFI)) using the GraphPad Prims software.

## 3. Results

### 3.1. LIMD2 is Specifically Expressed in PTC Cells

**Figure 1:**
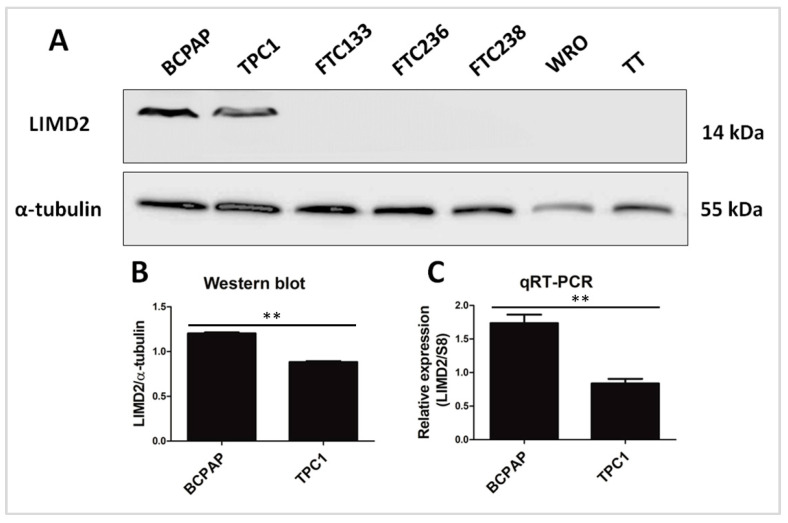
LIMD2 expression in thyroid cell lines. (**A**) Representative image of Western blot analysis shows that LIMD2 is exclusively expressed in papillary (BCPAP and TPC1), while it is not expressed in follicular (FTC133, FTC236, FTC238, and WRO) and medullary (TT) thyroid carcinoma cell lines. The α-tubulin was used as a loading control; (**B**) Densitometry measurement of LIMD2 expression. The results are the ratio of LIMD2/α-tubulin from three independent experiments. Each column represents the mean ± SD; (**C**) *LIMD2* expression in BCPAP and TPC1 cell lines. S8 was used as an internal control. Each column represents the mean ± SD of three experiments performed in triplicate. ** *p* < 0.01.

Western blot analysis revealed that LIMD2 (14 kDa) was specifically expressed in PTC cell lines (BCPAP and TPC1 cell lines). Remarkably, LIMD2 was expressed at higher levels in BRAF V600E-positive cells (BCPAP) than in RET/PTC1-positive cells (TPC1) (Figure 1A).

The results of three independent Western blot analyses are graphically represented in Figure 1B. The qRT-PCR confirmed that LIMD2 expression was higher in the cell line harboring BRAF V600E than in the cells harboring RET/PTC fusion (Figure 1C).

The analysis of copy number state showed that the TPC1 cell line presented a copy number gain of *LIMD2* gene, while the BCPAP cell line presented normal copy number state (Supplementary Table S1).

### 3.2. CRISPR/Cas9 System Successfully Promoted the LIMD2 KO in Thyroid Carcinoma Cell Lines

To evaluate the editing efficiency of each sgRNAs (Table 3), the cells were independently transfected and prescreened for high editing efficiency and specificity. Sequencing of PCR product showed 99% identity with sequence assembled to GRCh38.p12 on chromosome 17 (location NC_000017.11), which corresponded to LIMD2 gene.

**Figure 2:**
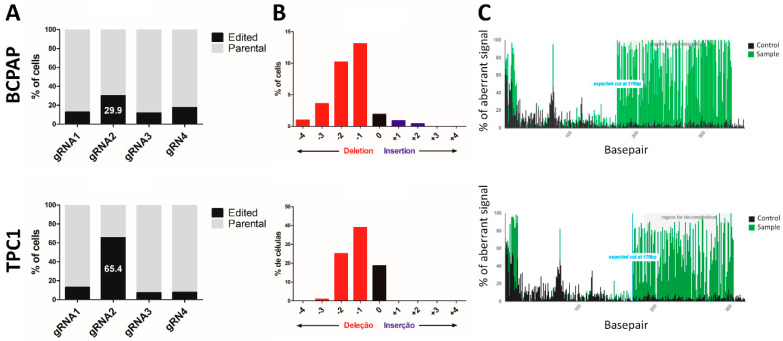
CRISPR/Cas9 introduced frameshift insertion/deletion (indel) mutations of LIMD2. (**A**) tracking of indels by decomposition (TIDE) decomposition algorithm analysis showing high editing efficiency of four different gRNAs. The gRNA2 exhibited the highest editing efficiency rate for BCPAP (29.9%) and TPC1 (65.4%) cells; (**B**) The indel spectrum within ±4 bp from predicted gRN2A cleavage site. The majority of edited cells presented deletion of a single nucleotide; (**C**) TIDE decomposition analysis showing the detection of mixed peaks in the Sanger sequence reads. The aberrant signal in non-edited (black) and edited cells (green) and the expected cleavage site at 170 bp (blue line).

The DNA sequences from a mixed pool of parental (untreated cells) and a mixed pool of CRISPR edited cells were decomposed and aligned using the TIDE tool, and the results showed that the four gRNAs were able to edit the gene LIMD2, but with different efficiency rates. The gRNA2 showed the highest efficiency rate for both cell lines, and therefore was selected for further analysis (Figure 2A). The gRNA2 was able to introduce small insertions and deletions (indels) at the cleavage site, which could cause frameshift mutations or premature stop codons (Figure 2B). Analysis of the raw sequencing data confirmed that the Cas9 introduced a cleavage at the 170 pb position (Figure 2C).

Next, we selected single cell clones from KO cell pools via the limiting dilution method. Seven clones of BCPAP and six clones of TPC1 were obtained from each pool. The sequence of each clone was aligned to the canonical amino acid sequence of LIMD2 using the AliView version 1.3 software [15]. Because the LIMD was the main domain affected in the LIMD2-edited clones, only LIM consensus sequence was shown (residues 38 to 98). The sequence alignment showed that all seven edited clones shared the same single-nucleotide variation consisting of 1 bp deletion from the residue 43, which corresponded to second cysteine (Cys43) that binded to a zinc ion within the first zinc-finger domain. Although the CRISPR/Cas9 introduced a reading frameshift mutation, it did not generate a premature stop codon. Importantly, 1 bp deletion, present in edited clones, shifted the way the LIMD2 sequence was read from residue 43 and likely resulted in a folding change of the LIM domain and the loss of function of LIMD2 protein (Supplementary Figure S1). Considering that all clones had the same mutation and the use of additional clones would not provide additional information, one clone of each edited cell line (clone 1) was selected for the further studies.

### 3.3. No Altered Sequence was Found in any of the Potential Off-Target Genes

We searched for potential off-target sites in the human genome that might be recognized by the LIMD2 gRNA2. We identified five potential candidate sites whose sequences were conserved in the seed region but contained three base mismatches in the 5′ end. However, no gene was mapped to the specific region on chromosomes 15, X, and 3 (Table 4). Sequencing analysis revealed that mutations were not detected at any of the putative off-target sites located within *COL19A* (6q13) and *PPAR4* (13q12.12) genes (Supplementary Figure S2), suggesting that the CRISPR/Cas9 system was highly specific.

### 3.4. CRISPR/Cas9 System Promoted Functional KO of LIMD2

**Figure 3:**
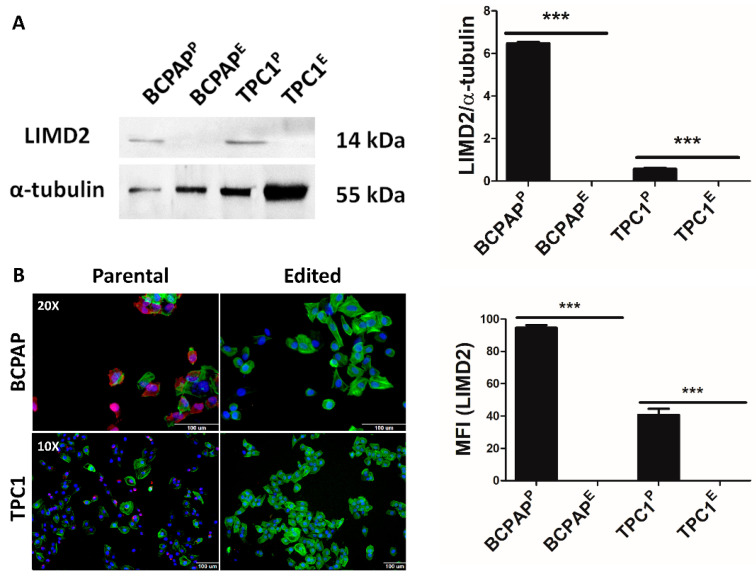
CRISPR/Cas9 promoted complete knockout (KO) of LIMD2 protein. (**A**) Representative Western blot images and graphic representation for LIMD2 shows complete KO in edited cells (BCPAP^E^ and TPC1^E^), while it is expressed in parental or untreated cells (BCPAP^P^ and TPC1^P^). Each column represents the mean ± SD of three experiments performed in triplicate; (**B**) Representative images of the high-content screening analysis and graphic representation confirmed the absence of LIMD2 (pink) expression in both cell line. Green, phalloidin-labeled F-actin and blue, nucleus. Scale bar of 100 μm. MFI, medium of fluorescence intensity. *** *p* < 0.001.

Using an antibody that was able to recognize the C-terminal region of LIMD2 (residues 105–126, which are located downstream of the editing site), we confirmed that LIMD2 expression was absent in edited cells (BCPAP^E^ and TPC1^E^), while it was expressed in the untreated cells, henceforward called parental cells (Figure 3A).

Data of three independent assays were quantified and graphically represented (Figure 3A), suggesting that the CRISPR/Cas9 system successfully promoted functional KO of the target gene. The HCS analysis confirmed the absence of LIMD2 in edited cells and also showed differences in cell morphology (Figure 3B). F-actin staining advocated that LIMD2 KO induced changes in actin cytoskeleton remodeling, revealing a likely role of LIMD2 in cell invasion (Figure 3B).

### 3.5. LIMD2 KO Restored Thyroid Cell Polarity and Mitochondria Morphology

**Figure 4:**
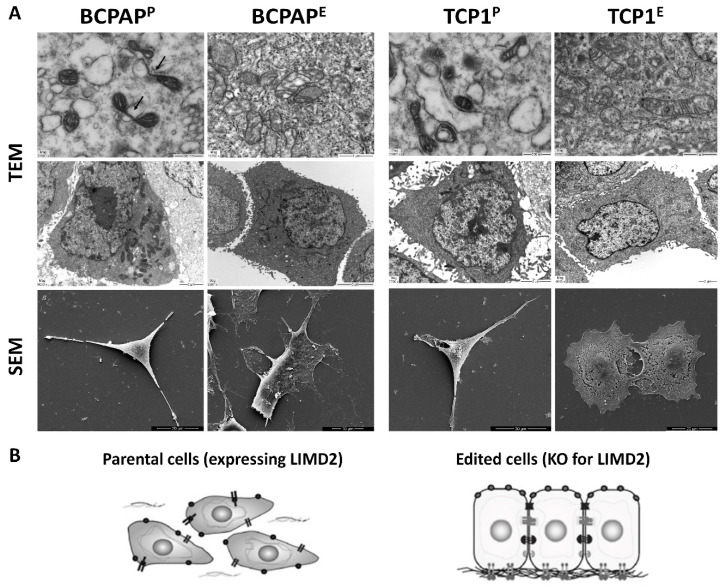
(**A**) Representative images of transmission electron microscopy (TEM) and scanning electron microscopy (SEM). Parental cells (BCPAP^P^ and TPC1^P^) presented loss of apical-basal polarity and concentration of mitochondria in one pole of the cells. The presence of small and circular mitochondria suggests mitochondrial fission. Vacuoles and mitochondrial fission are showed (arrow). Remarkably, the apical-basal polarity and mitochondria morphology are somewhat restored in LIMD2-edited cells (BCPAP^E^ and TPC1^E^); (**B**) Schematic model of parental (expressing LIMD2) and edited cells (complete KO of LIMD2).

The ultrastructural morphology analysis showed that parental cells lost the apical-basal polarity commonly observed in epithelial cells and the cell polarity was partially restored in LIMD2-edited cells (Figure 4). Similar data was observed in the HCS analysis (Figure 3B). These differences in cell shape are likely associated with differences in specialized function. In addition, parental and LIMD2-edited cells display distinct mitochondrial morphology. A higher number of vacuoles and mitochondrial fission arrest phenotypes, which resulted in elongated interconnect organelles, was observed in parental cells. Mitochondria were observed in one of the cell poles in parental cells. In the LIMD2-edited cells, a more homogenous mitochondrial distribution was observed in the cytoplasm. Additionally, mitochondria with normal structures of membranes and crest and normal size were observed (Figure 4).

Taken together, our data suggest that LIMD2 might promote morphology changes in PTC cells, such as mitochondria fission and redistribution to the leading edge of the cells, and to likely sustain the bioenergetic demands during invadopodia [17].

### 3.6. LIMD2 KO Reduces the Cell Invasion

**Figure 5:**
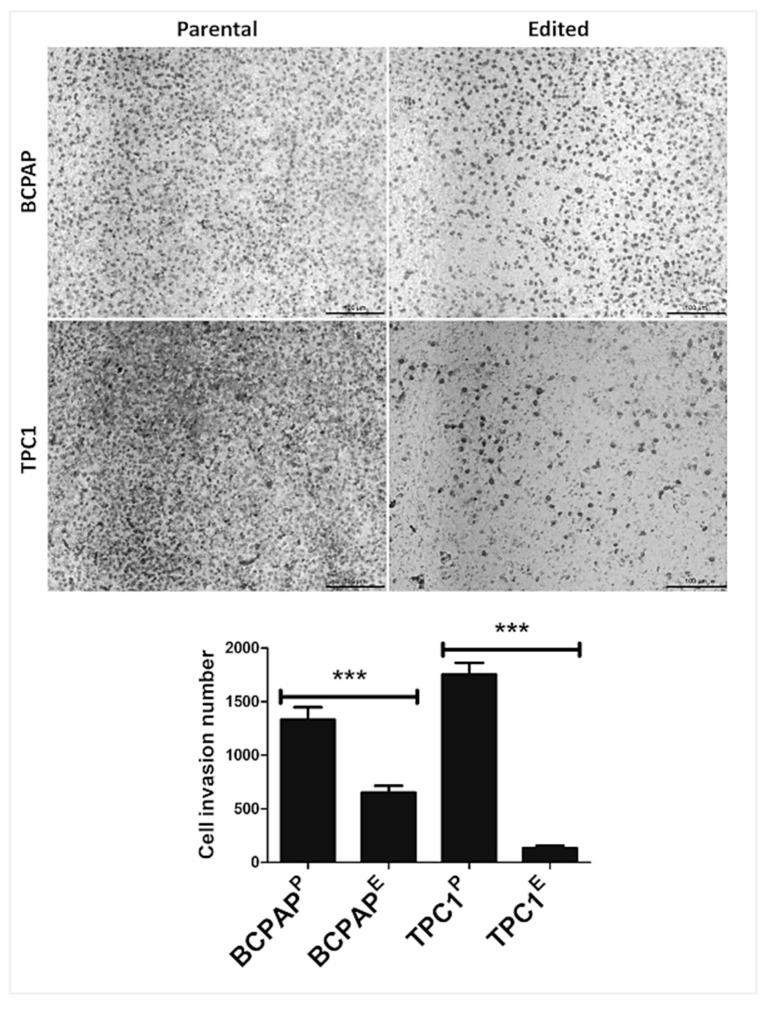
LIMD2 knockout in BCPAP and TPC1 cells showed reduced in vitro cell invasion. Representative images showed a reduced number of invasive cells in LIMD2-edited cells (BCPAP^E^ and TPC1^E^) as compared with parental cell lines (BCPAP^P^ and TPC1^P^). The data of three independent experiments showed that the percentage of invasive cells in BCPAP^P^ (67%) and TPC1^P^ (88%) significantly reduced in BCPAP^E^ (35%) and TPC1^E^ (7%). *** *p* < 0.001.

The invasion rate was significantly higher in the parental cells (BCPA^P^ and TPC1^P)^ as compared with LIMD2-edited cells (BCPAP^E^ and TPC1^E^). Of note, a more prominent result was observed for TPC1 cells (Figure 5).

### 3.7. Decreased Serine/Threonine Kinase Phosphorylation in LIMD2 KO Cells

**Figure 6:**
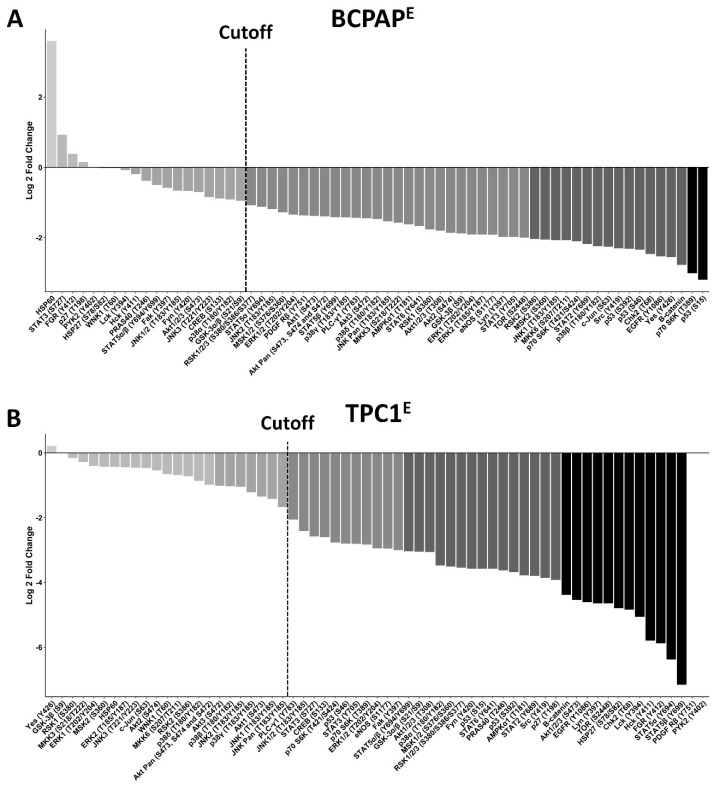
LIMD2 knockout significantly reduced phosphorylation of nearly 60 kinases. The Human Phospho-MAPK and Human Phospho-Kinase Antibody Array Kit were assessed in LIMD2-edited cells (BCPAP^E^ and TPC1^E^) and parental cells. (**A**) BCPAP^P^; (**B**) TPC1^P^. Log graph based on the fold change of all kinases analyzed in the proteome profiler array, showing that the *LIMD2* KO reduced the phosphorylation levels of the majority of kinases analyzed.

Several members of the MAPK superfamily, which consist of the extracellular signal-regulated protein kinases (ERKs), the c-Jun N-terminal kinases (JNKs), and the p38 family of kinases, showed reduced levels of phosphorylation upon LIMD2 KO in thyroid cells (Figure 6).

In order to identify kinases with robust changes in magnitude of phosphorylation, we used a fold change (FC) cutoff of 1.3 and 0.7. These changes in kinase activity, with similar or smaller orders of magnitude, have previously been considered to be sufficient to trigger biologically relevant changes. Kinases with increased (FC of 1.30) or decreased (FC of 0.70) phosphorylation are shown. The fold change was transformed log2 and plotted in a histogram (Figure 6). Although the mean phosphorylation level was more drastically diminished in TPC1^E^, the number of kinases whose phosphorylation status changed upon LIMD2 KO was higher in BCPAP^E^ cells (Figure 6).

The phosphorylation status of over 40 kinases decreased and very few increased upon LIMD2 KO in BCPAP^E^ (Figure 6) and TPC1^E^ (Figure 6) cells. Remarkably, LIMD2 KO completely abolished the phosphorylation of PDGFRβ and PYK2 proteins in the TPC1 cells (Figure 6).

**Figure 7:**
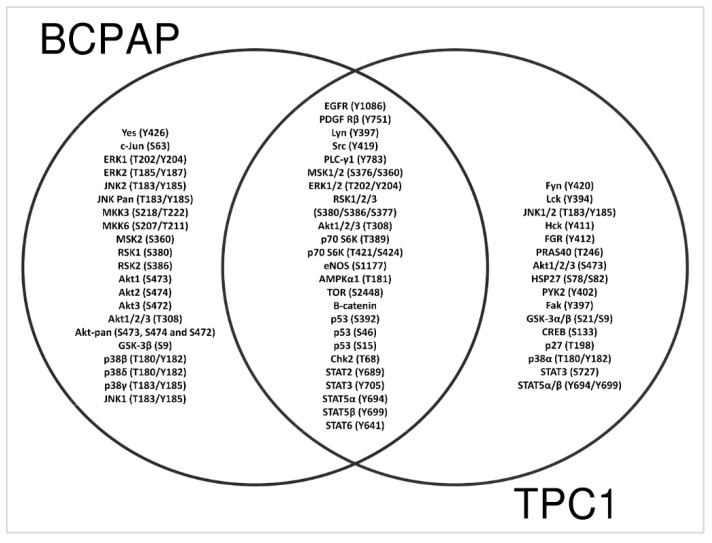
Venn diagram showing the kinases that were modulated in both cells (intersection) or either in BCPAP or TPC1 LIMD2-edited cells.

The Venn diagram shows the distribution of unique and shared set of kinases that were differentially phosphorylated in the LIMD2-edited cells. The overlap intersection region denotes a list of proteins whose phosphorylation status changed in both LIMD2-edited cells, including ERK1/2, AKT1/2/3, TP53, members of STAT family, and β-catenin. The two disjoint regions denote kinases whose phosphorylation status changed exclusively in BCPAP or in TPC1 LIMD2-edited cells (Figure 7).

**Figure 8:**
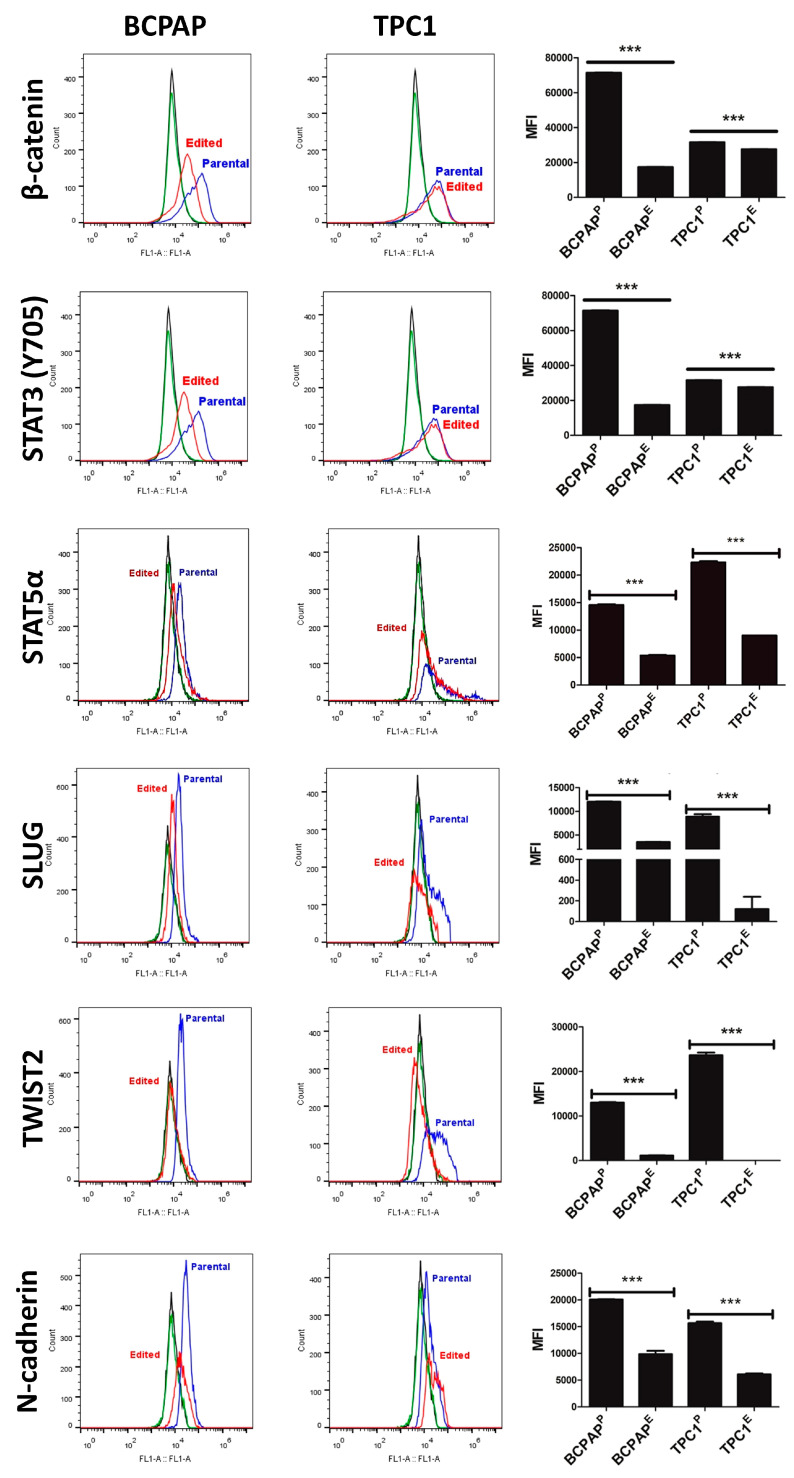
Results of immunodetection of β-catenin, STAT3 (Y705), STAT5α, SLUG, TWIST2, and N-cadherin by flow cytometry. Results show a significant downregulation of all analyzed proteins in both edited cells. Flow cytometry analyses show cells not incubated with primary and secondary antibody (histogram in black), cells exclusively incubated with secondary antibody (histogram in green), and cells incubated with both primary and secondary antibodies (histogram in red). There were 10,000 events analyzed in duplicate. *** *p* < 0.001.

**Figure 9:**
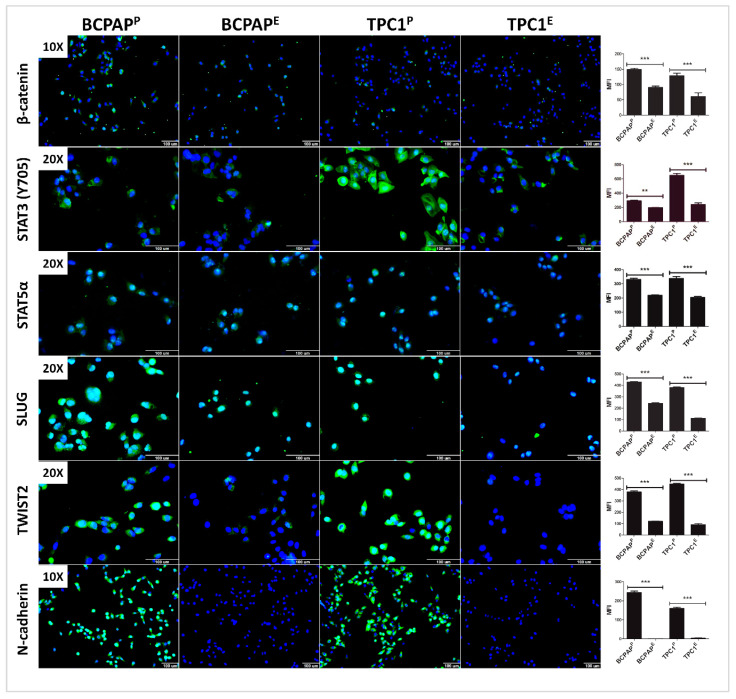
Results of immunodetection of β-catenin, STAT3 (Y705), STAT5α, SLUG, TWIST2, and N-cadherin by high-content screening. Results show a significant downregulation of all analyzed proteins in both edited cells and the absence of nuclear immunodetection of STAT3 (Y705) and SLUG in BCPAP^E^ and TPC1^E^ cell lines. Scale bar of 100 μm. *** *p* < 0.001 and ** *p* < 0.01.

Flow cytometry (Figure 8) and HCS (Figure 9) confirmed that LIMD2 KO reduced the expression of β-catenin, STAT3 (Y705), and STAT5α levels in both cell lines, reinforcing the results observed in the proteome profile array.

### 3.8. LIMD2 KO Reduces the Expression of Proteins Associated with Epithelial Mesenchymal Transition

Because STAT3 and several kinases were found dephosphorylated, upon LIMD2 KO mediate the phosphorylation of proteins previously related to EMT, we next validated the expression of master regulators of EMT. Flow cytometry and HCS revealed that LIMD2 KO reduced the expression and nuclear localization of SLUG and TWIST2 in both cell lines, while they were highly expressed in the cytoplasm and the nucleus of parental cells (Figure 8 and Figure 9). Because the diminished expression of N-cadherin is a hallmark of EMT, which is regulated by a complex network of signaling pathways and transcription factors such as SLUG and TWIST, we next investigated the N-cadherin expression. As anticipated, LIMD2 KO reduced the expression levels of N-cadherin (Figure 8 and Figure 9). Different from expected, the expression levels of N-cadherin diminished in LIMD2 KO cells. Taken together, these results strongly suggest that LIMD2 plays a role in EMT of papillary thyroid carcinomas.

### 3.9. KO of LIMD2 Reduced Phosphorylation of Proteins that Function as a Guardian of the Genome

**Figure 10:**
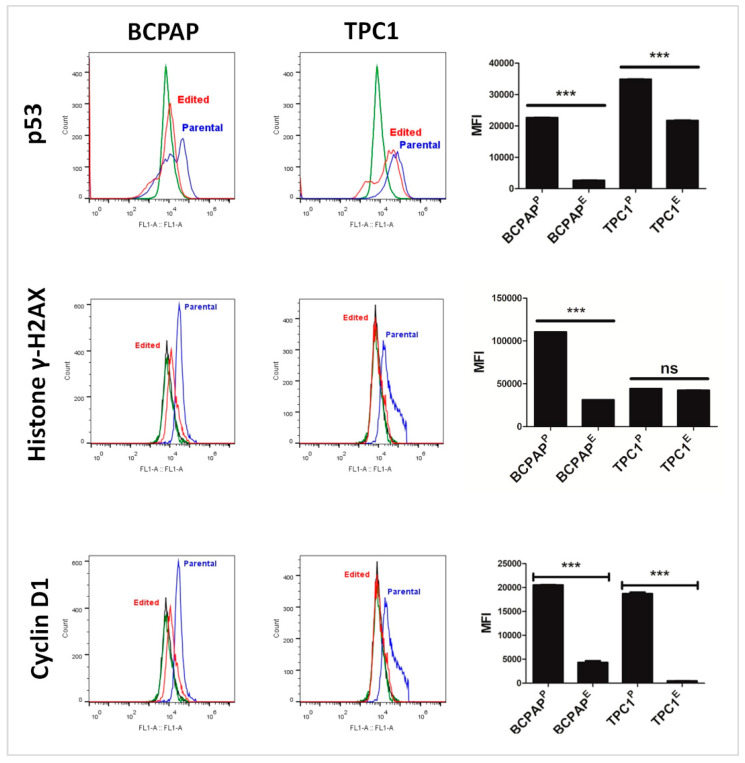
Results of immunodetection of p53, histone γ-H2AX, and cyclin D1 by flow cytometry. Results show a significant downregulation of all analyzed proteins in both edited cells. Flow cytometry analyses show cells not incubated with primary and secondary antibodies (histogram in black), cells exclusively incubated with secondary antibody (histogram in green), and cells incubated with both primary and secondary antibodies (histogram in red). There were 10,000 events analyzed in duplicate. *** *p* < 0.001, non-significant statistical difference (ns).

Considering that phosphorylation of p53 (S392) was related to genomic instability, we analyzed the phosphorylation levels of this residue using flow cytometry. *LIMD2* KO reduced the phosphorylation of p53 (S392) in both cell lines (Figure 10), suggesting that the LIMD2 likely increased genomic instability. As DNA damage, such double-strand breaks and eroded telomeres initiate a signaling cascade designed to arrest the cell cycle through checkpoint activation and histone H2AX phosphorylation on serine 139 (γ-H2AX), we next analyzed the expression γ-H2AX. A significant reduction in γ-H2AX phosphorylation was observed in BCPAP^E^ cells, suggesting that the cell cycle might be resumed. However, significantly statistical differences were not observed between the phosphorylation levels of TPC1^P^ and TPC1^E^ cells. The expression of cyclin D1 was reduced in both edited cells (Figure 10).

## 4. Discussion

Previously, LIMD2 has been found to be overexpressed in many cancer types, including PTC, bladder, melanoma, breast, and non-small cell lung cancer, while its expression is negligible in normal tissues [6,7,8]. It has also been demonstrated that LIMD2 levels correlated with the metastatic process in PTCs and invasiveness and poor prognosis in patients melanoma, bladder, and non-small cell lung cancer [6,7,8,18,19]. In vitro analysis have demonstrated that LIMD2 promoted cell migration and invasion and its silencing suppressed the proliferation and invasion [8,18,19]. Although these observations indicate that LIMD2 is involved in the first steps of the metastatic process, the signaling pathways that orchestrate these steps are undetermined. 

Aiming at understanding the role of *LIMD2* in the metastatic process of thyroid carcinomas, we assessed the expression of LIMD2 in thyroid cell line models for distinct cancer subtypes. LIMD2 was found to be exclusively expressed in PTC cell lines with BRAF V600E (BCPAP) or RET/PTC fusion (TPC1) and its expression could be traced to the magnitude of MAPK signaling, reinforcing our previous results [7].

To explore the role of LIMD2 in the biological process related to metastasis in thyroid models, we used the CRISPR/Cas9 gene-editing system to disrupt *LIMD2* gene sequence and abolish LIMD2 protein expression BCPAP and TPC1, and presented endogenous expression of LIMD2.

We showed that the four tested gRNAs were able to produce pools with short insertions/deletions (indels), regardless of whether *LIMD2* was disrupted by exons 4 or 5 mutations. Because the gRNA2 mixed poll showed the highest editing efficiency rate, it was selected to obtain a single-cell-derived clone. Although the highest editing efficiency of gRNA was initially verified in TPC1 cells, which was attributed to the different genetic background, all the single cell clones selected for both cell lines presented a 1 bp deletion. The CRIPS/Cas9 introduced, in most cells, a frameshift mutation within exon 4, which ultimately likely caused protein KO via premature stop codon.

Next, we showed that we readily achieved complete inactivation of LIMD2 in TPC1 and BCPAP clones, as the absence of LIMD2 protein was seen in CRISP/Cas9-edited clones.

*LIMD2* KO by CRISPR/Cas9 significantly inhibited cellular processes associated with initial steps of metastases such as cell migration and invasion in PTC cells. To ascertain that the phenotypic alterations observed were not due to off-target effects, we sequenced the potential off-targets and found no mutations. Thereby, we established an efficient model for functional studies.

The ultrastructural analysis showed that *LIMD2* deficiency in PTC cells restored cell epithelial polarity and mitochondria morphology along with actin reorganization fiber. Because this more differentiated phenotype observed in LMD2-edited cells came along with reduced migration and invasion, there were two fundamental questions that needed to be answered. What are the underlying interwoven networks by which LIMD2 acts in PTC models? Are the EMT markers also modulated following LIMD2 KO?

Regarding the underlying network, using fibroblast cells as a model, it was shown that LIMD2 binds to and activates the activity of integrin-linked kinase (ILK) [8]. It is acknowledged that ILK is a receptor-proximal protein which plays a central role in linking extracellular signaling that regulates processes that are crucial to tumor progression, including the regulation of cell shape, motility, growth, survival, differentiation; modulates actin rearrangement and angiogenesis; and inhibits anoikis and apoptosis as well as gene expression. It is known that ILK exerts its biological functions through distinct signaling pathways such as PI3K/AKT/mTOR, GSK3β, NF-κB, Hippo, ERK, VEGF, STAT3, Wnt1, and Snail1 [20]. Therefore, it would be crucial to assess the level of ILK in BCPAP and TPC1 cells. Because its expression was previously assessed in several thyroid cancer cell lines, but it was expressed at very low levels in TPC1 and BCPAP cells, the role of ILK in migration, invasion, and modulation of EMT-related proteins was not further investigated in these cells by the authors [21].

In this study, we demonstrated that LIMD2 KO significantly reduced phosphorylation of nearly 60 kinases. Selected kinases such as EGFR, PDGK, Src, ERK1/2, Akt1/2/3, mTOR, p70S6k, AMPKα, P53, β-catenin, and several members of the STAT family showed reduced phosphorylation at particular phosphorylation sites in both cells, as shown in the intersection of the Venn diagram. Although a priori, the Venn diagram results suggest an inconsistency, the differences in the expression levels of the ERK, JNK, and AKT and their isoforms (ERK1/2, JNK1/2, and AKT1/2) are likely due to the fact that the antibodies that recognized more than one isoform of these kinases, as well as different phosphorylation sites, bind to the targets more efficiently than those able to bind to only one isoform or phosphorylation site. Whether there is a direct interaction between LIMD2 with specific binding sites on kinases in PTC cancer cells, which in turn trigger migration and invasion, needs to be determined.

Importantly, flow cytometry and HCS strengthened the kinase phosphorylation profiles data, as *LIMD2* KO reduced significantly the β-catenin and STAT5α expression and STAT3 (Y705) phosphorylation in both edited cell lines.

Intriguingly, MAPK family members such as c-Jun and p38β, δ, and γ are particularly affected in BCPAP cells that harbor BRAF V600E, TP53, and TERT mutations and a more aggressive phenotype. Other kinases (p38α and CREB) are specifically associated with TPC1 that harbor *RET/PTC* fusion and a less aggressive phenotype. These data can explain, at least in part, the different biological behavior of these two PTC cell lines.

Taken together, these results suggest that LIMD2 activates kinases that favor the trans-differentiation of the epithelial phenotype to a mesenchymal phenotype and promotes cancer metastasis in a cell dependent context. However, many intrinsic (e.g., gene mutations) and extrinsic (e.g., growth factors) signals are able to initiate or sustain the EMT phenotype. For example, it has been demonstrated that the phosphorylation of a conserved tyrosine residue (Y705) of STAT3 and STAT5 can be activated by upstream kinases such as epidermal growth factor receptor (EGFR), platelet-derived growth factor receptor (PDGFR), and non-receptor tyrosine kinases such as Janus kinase (JAK). Subsequent phosphorylation STAT3 dimers translocate into the nucleus, where they bind to specific DNA response in the promoter of transcription factors considered to be master regulators of EMT expression, such as SLUG and TWIST [22]. A connection has also been shown among STAT3 phosphorylation, regulation of GSK3β, and the expression of β-catenin and TWIST [21,23]. Finally, STAT3 also regulates the expression of tumor suppressor genes such as TP53 and proliferating genes such as cyclin D1 [24,25,26,27,28,29].

As aforementioned, LIMD2 KO diminished the phosphorylation of STAT3 and GSK3β, and reduced the expression of β-catenin, SLUG, TWIST2, and N-cadherin. *LIMD2* KO also reduced the phosphorylation at three different sites of p53, including S392 which is the target site for different kinases, including p38 [30]. In this sense, diminished phosphorylation of different isoforms of p38 can explain the reduced phosphorylation of p53 (S392).

Because p53 accumulation and phosphorylation maintains genetic stability through cell cycle arrest, apoptosis, senescence, and autophagy [31], we next measured the expression of target genes associated with the cell cycle such as *CCND1,* as well as, evaluated the protective role of p53 against DNA damage in the S-phase detectable by γ-H2AX positivity. LIMD2 KO reduced the phosphorylation γ-H2AX in BCPAP cells [32,33]. These data suggest, for the first time, that LIMD2 increases the proteins associated with DNA damage checkpoint via replicative stress, and therefore increases genomic instability. The phosphorylation of γ-H2AX is not reduced in TPC1^E^ cell line, suggesting that unlike RET/PTC1, BRAF V600E mutation likely plays a significant role in genetic instability and that LIMD2 may cooperate to maintain a more aggressiveness phenotype and PTC progression though modulation of p53 and γ-H2AX proteins.

## 5. Conclusions

Our findings suggest that STAT3 may regulate p53 or even p53 and STAT3 may have a crosstalk through the combined regulation of other kinases such as RAF/RAS/MAPK and PI3K/AKT or p38 MAPK, which are in line with previous observations [23,30]. Therefore, we propose that an important primary function of LIMD2 is to regulate phosphorylation of kinases that are associated with EMT, thus, promoting invasion in PTC. LIMD2 could contribute, through cooperation with different kinases, to the increased genomic instability that eventually promotes PTC progression. Indeed, LIMD2 is a candidate biomarker of tumor progression that could be used for PTC in liquid biopsies or novel therapeutic strategies.

## Figures and Tables

**Table 1:** Thyroid carcinoma cell lines used in this study.

Cell Line	Histological Type	Medium	Source	Mutation Identified ^1^
BCPAP	PTC	RPMI 1640 medium, supplemented with 5% FBS	DSMZ Cat No. ACC 273	BRAF V600E, TP53 D258Y, TERT c.-124C>T and c.-125C>T
TPC1	PTC	RPMI 1640 medium, supplemented with 10% FBS	João R. Maciel, UNIFESP, SP, Brazil	RET/PTC1, TERT c.-124C>T
FTC133	FTC	DMEM and Ham’s F12 (1:1) medium, supplemented with 10% FBS	ECACC cat no. 94060901	NF1 C167*, PTEN R130*, TP53 R273H, TERT c.-124C>T, RB1*, MSH6*
FTC236	FTC	DMEM and Ham’s F12 (1:1) medium, supplemented with 10% FBS, 1 mU/mL TSH, and 10 µg/mL insulin	ECACC cat. no. 06030202	PTEN R130*, TP53 R273H, TERT c.-124C>T, RB1*, MSH6* and others
FTC238	FTC	DMEM and Ham’s F12 (1:1) medium, supplemented with 10% FBS	ECACC cat no. 94060902	PTEN R130*, TP53 R273H, TERT c.-124C>T, RB1*, MSH6*
WRO	FTC	DMEM medium, supplemented with 10% FBS	Alfredo Fusco, Università degli Studi di Napoli Federico II, Italy	TP53 P223L, BRAF wild type, TERT promoter wild type
TT	MTC	F-12K medium, supplemented with 10% FBS	ECACC cat no. 92050721	RET C634W

^1^ Mutations characterized by Landa et al. (2019) [11]. PTC, papillary thyroid carcinoma; FTC; follicular thyroid carcinoma; MTC, medullary thyroid carcinoma.

**Table 2:** Antibodies specifications and working solutions used in this study.

Target	m/pAb	Specie	Source	Cat. Number	Dilution	Assay
LIMD2 ^1^	pAb	Rabbit	Peng et al. [8]	-	1: 1000	WB
LIMD2 ^2^	pAb	Rabbit	Abcam	Ab221110	1:33	WB, HCS
Α-tubulin	mAb	Mouse	Sigma-Aldrich	T9026	1:10,000	WB
F-actin ^3^	-	-	Thermo Fisher Scientific	A12379	1:300	HCS
Histone-γH2AX	pAb	Rabbit	Novus Biologicals	NB100-384	1:100	FC
P53	pAb	Rabbit	Abcam	ab131442	1:100	FC
β-catenin	mAb	Mouse	Santa Cruz Biotechnology	Sc-376841	1:100	FC, HCS
STAT3 (Y705)	pAb	Rabbit	R&D Systems	AF4607	1:100	FC, HCS
STAT5α	mAb	Mouse	Santa Cruz Biotechnology	Sc-166479	1:200	FC, HCS
SLUG	pAb	Rabbit	Santa Cruz Biotechnology	sc-15391	1:100	FC, HCS
TWIST2	mAb	Mouse	Abcam	Ab50887	1:50	FC, HCS
N-cadherin	mAb	Mouse	Dako	M3613	1:100	FC, HCS
FITC-conjugated goat anti-rabbit IgG	pAb	Goat	Thermo Fisher Scientific	AB42978	1:1000	FC, HCS
FITC-conjugated goat anti-mouse IgG	pAb	Goat	Thermo Fisher Scientific	AB2533946	1:10,000	FC, HCS

WB, Western blot; FC, flow cytometry; HCS, high-content screening. ^1^ LIMD2 antibody developed by Peng et al. [8] was used to screen the LIMD2 expression in cell lines derived from different histological types of thyroid cancer. This antibody is now commercialized by Merck (MABC1168) and recognizes the full-length human LIMD2. ^2^ LIMD2 antibody Ab221110 was used to confirm the LIMD2 knockout in both edited cells, since it recognized the amino acid residues 105–126 (C-terminal region) comprised within the edited region. ^3^ F-actin was detected using the FITC-conjugated phalloidin mycotoxin.

**Table 3:** Sequences of the CRISPR RNA (crRNA) used in this study.

gRNA	crRNA	Off-Targets	Exon
1	GGCAACTACGACGAGGGGTTTGG	0	5
2	CCAGAAGACCGTGTACCCCATGG	5	4
3	GTTTGGCCGCAAGCAGCACAAGG	2	5
4	AGCAAAGGCAACTACGACGAAGG	2	5

The crRNA binding sites are upstream to the target region of LIMD2 with 3 bp protospacer adjacent motif (PAM) recognized by the Cas9, showing the number of predicted off-targets and the target exon. Sequences identified in reverse strand of gene *LIMD2*. The PAM (NGG) sequence is underline.

**Table 4:** Potential off-target sites in the human that might be recognized by LIMD2 gRNA2.

Sequence	PAM	Coordinate	Gene	*Locus*
CCAAAAGACCGGATACCCCA	CGG	chr15[74825883]	-	-
CCAGAAGACCGTGCAGCCCC	TGG	chrX[132498708]	-	-
GCAGAAGTCCGTGAACCCCA	TGG	chr3[153321832]	-	-
CCAGAAGTCTGTGTTCCCCA	TGG	chr13[24452558]	*PARP4*	13q12.12
CCAGGAGACCCTGGACCCCA	AGG	chr6[70199715]	*COL19A1*	6q13

Nucleotides in red show the mismatches.

## Supplementary Materials

The following are available online at https://www.mdpi.com/2073-4409/9/11/2522/s1, Figure S1: Results of off-target analyses, Figure S2: DNA sequencing results of the isolated clones, Table S1： Results of copy number variation (CNV) analysis.

## References

[1] Maksimovic S., Jakovljevic B., Gojkovic Z. (2018). Lymph node metastases papillary thyroid carcinoma and their importance in recurrence of disease. Med. Arch..

[2] Bohec H., Breuskin I., Hadoux J., Schlumberger M., Leboulleux S., Hartl D.M. (2019). Occult contralateral lateral lymph node metastases in unilateral N1b papillary thyroid carcinoma. World J. Surg..

[3] Pazaitou-Panayiotou K., Kaprara A., Chrisoulidou A., Boudina M., Georgiou E., Patakiouta F., Drimonitis A., Vainas I. (2005). Cerebellar metastasis as first metastasis from papillary thyroid carcinoma. Endocr. J..

[4] Haugen B.R., Alexander E.K., Bible K.C., Doherty G.M., Mandel S.J., Nikiforov Y.E., Pacini F., Randolph G.W., Sawka A.M., Schlumberger M. (2016). 2015 American Thyroid Association Management Guidelines for Adult Patients with Thyroid Nodules and Differentiated Thyroid Cancer: The American Thyroid Association Guidelines Task Force on Thyroid Nodules and Differentiated Thyroid Cancer. Thyroid.

[5] Tuttle R.M., Haugen B., Perrier N.D. (2017). Updated American Joint Committee on Cancer/Tumor-Node-Metastasis Staging System for Differentiated and Anaplastic Thyroid Cancer (Eighth Edition): What Changed and Why?. Thyroid.

[6] Cerutti J., Oler G., Michaluart P., Delcelo R., Beaty R., Shoemaker J., Riggins G. (2007). Molecular profiling of matched samples identifies biomarkers of papillary thyroid carcinoma lymph node metastasis. Cancer Res..

[7] Santos M.J.C.P., Bastos A.U., Costa V.R., Delcelo R., Lindsey S.C., Colozza-Gama G.A., Peng H., Rauscher F.J., Oler G., Cerutti J.M. (2018). LIMD2 Is Overexpressed in BRAF V600E-Positive Papillary Thyroid Carcinomas and Matched Lymph Node Metastases. Endocr. Pathol..

[8] Peng H., Talebzadeh-Farrooji M., Osborne M., Prokop J., McDonald P., Karar J., Hou Z., He M., Kebebew E., Orntoft T. (2014). LIMD2 is a small lim-only protein overexpressed in metastatic lesions that regulates cell motility and tumor progression by directly binding to and activating the integrin-linked kinase. Cancer Res..

[9] Araldi R.P., Cerutti J.M. (2018). Novel biotechnological opportunities in thyroid cancer metastasis based on LIMD2 differential expression. Trend Cancer Res. Chemother..

[10] Koch B.J., Ryan J.F., Baxevanis A.D. (2012). The diversification of the lim superclass at the base of the metazoa increased subcellular complexity and promoted multicellular specialization. PLoS ONE.

[11] Landa I., Pozdeyev N., Korch C., Marlow L.A., Smallridge R.C., Copland J.A., Henderson Y.C., Lai S.Y., Clayman G.L., Onoda N. (2019). Comprehensive genetic characterization of human thyroid cancer cell lines: A validated panel for preclinical studies. Clin. Cancer Res..

[12] Rueden C.T., Schindelin J., Hiner M.C., DeZonia B.E., Walter A.E., Arena E.T., Eliceiri K.W. (2017). ImageJ2: ImageJ for the next generation of scientific image data. BMC Bioinform..

[13] Oliveira M.N.L., Hemerly J.P., Bastos A.U., Tamanaha R., Latini F.R.M., Camacho C.P., Impellizzeri A., Maciel R.M.B., Cerutti J.M. (2011). The RET p.G533C mutation confers poredisposition to multiple endocrine neoplasia type 2A in a Brazilian kindred and is able to induce a malignant phenotype in vitro and in vivo. Thyroid.

[14] Rao X., Huang X., Zhou Z., Lin X. (2013). An improvement of the 2^(-delta delta CT) method for quantitative real-time polymerase chain reaction data analysis. Biostat. Bioinform. Biomath..

[15] Larsson A. (2014). AliView: A fast and lightweight alignment viewer and editor for large datasets. Bioinformatics.

[16] Moraes L., Zanchin N.I.T., Cerutti J.M. (2017). ABI3, a component of the WAVE2 complex, is potentially regulated by PI3K/AKT pathway. Oncotarget.

[17] Pettersen E.F., Goddard T.D., Huang C.C., Couch G.S., Greenblatt D.M., Meng E.C., Ferrin T.E. (2004). UCSF Chimera? A visualization system for exploratory research and analysis. J. Comput. Chem..

[18] Desai S.P., Bhatia S.N., Toner M., Irimia D., Chase C. (2013). Mitochondrial localization and the persistent migration of epithelial cancer cells. Biophys. J..

[19] Wang F., Li Z., Xu L., Li Y., Li Y., Zhang X., Wang Y., Liu D. (2018). LIMD2 targeted by miR‑34a promotes the proliferation and invasion of non‑small cell lung cancer cells. Mol. Med. Rep..

[20] Zhang F., Qin S., Xiao X., Tan Y., Hao P., Xu Y. (2019). Overexpression of LIMD2 promotes the progression of non-small cell lung cancer. Oncol. Lett..

[21] Zheng C., Hu H., Hong P., Zhang Q., Xu W.W., He Q., Li B. (2019). Significance of integrin-linked kinase ( ILK ) in tumorigenesis and its potential implication as a biomarker and therapeutic target for human cancer. Am. J. Cancer Res..

[22] Shirley L.A., McCarty S., Yang M.C., Saji M., Zhang X., Phay J., Ringel M.D., Chen C.S. (2016). Integrin-linked kinase affects signaling pathways and migration in thyroid cancer cells and is a potential therapeutic target. Surgery.

[23] Li B., Huang C. (2017). Regulation of EMT by STAT3 in gastrointestinal cancer (Review). Int. J. Oncol..

[24] Liang F., Ren C., Wang J., Wang S., Yang L., Han X., Chen Y., Tong G., Yang G. (2019). The crosstalk between STAT3 and p53/RAS signaling controls cancer cell metastasis and cisplatin resistance via the Slug/MAPK/PI3K/AKT-mediated regulation of EMT and autophagy. Oncogenesis.

[25] Medici D., Hay E., Olsen B. (2008). Snail and Slug promote epithelial-mesenchymal transition through B-catenin-T-cell factor-4-dependent expression of transforming growth fator-B3. Mol. Biol. Cell.

[26] Visciano C., Liotti F., Prevete N., Cali’ G., Franco R., Collina F., de Paulis A., Marone G., Santoro M., Melillo R.M. (2015). Mast cells induce epithelial-to-mesenchymal transition and stem cell features in human thyroid cancer cells through an IL-8–Akt–Slug pathway. Oncogene.

[27] Buehler D., Hardin H., Shan W., Montemayor-Garcia C., Rush P., Asioli S., Chen H., Lloyd R. (2013). Expression of epithelial-mesenchymal transition regulators SNAI2 and TWIST1 in thyroid carcinomas. Mod. Pathol..

[28] Gasparotto D., Polesel J., Marzotto A., Colladel R., Piccinin S., Modena P., Grizzo A., Sulfaro S., Barzan L., Doglioni C. (2011). Overexpression of TWIST2 correlates with poor prognosis in head and neck squamous cell carcinomas. Oncotarget.

[29] Bremnes R., Veve R., Hirsch F., Franklin W. (2002). The E-cadherin cell–cell adhesion complex and lung cancer invasion, metastasis, and prognosis. Lung Cancer.

[30] Hajra K., Chen D., Fearon E. (2002). Advances in brief the SLUG zinc-finger protein represses E-cadherin in breast cancer 1. Cancer Res..

[31] Cox M.L., Meek D.W. (2010). Phosphorylation of serine 392 in p53 is a common and integral event during p53 induction by diverse stimuli. Cell. Signal..

[32] Maclaine N.J., Hupp T.R. (2009). The regulation of p53 by phosphorylation: A model for how distinct signals integrate into the p53 pathway. Aging.

[33] Sharma A., Singh K., Almasan A. (2012). Histone H2AX phosphorylation: A marker for DNA damage. Methods Mol. Biol..

