# Supplementary material for: LIMD2 Regulates Key Steps of Metastasis Cascade in Papillary Thyroid Cancer Cells via MAPK Crosstalk
Source: Cells. 2020 Nov 23;9(11):2522. doi: 10.3390/cells9112522 (PMC7700534; doi:10.3390/cells9112522)
Supplement: Supplementary file 1 [file cells-09-02522-s001.zip › supplementary final/1. Supplementary Table S1.docx]

**Table S1.** Results of copy number variation (CNV) analysis

| PBMC | Ct (*LIMD2*) | Ct (*ACTB*) | ΔCt | ΔΔCt | $\boldsymbol{2}^{\boldsymbol{-}\boldsymbol{\Delta\Delta Ct}}$ |
| --- | --- | --- | --- | --- | --- |
| Sample 1 | 22.870 | 22.975 | -0.105 | -0.055 | 1.025 |
| Sample 1 | 22.834 | 22.975 | -0.141 | -0.091 | 1.065 |
| Sample 1 | 22.532 | 22.666 | -0.134 | -0.084 | 1.060 |
| Sample 2 | 23.568 | 23.564 | 0.004 | 0.053 | 0.963 |
| Sample 2 | 23.092 | 23.841 | -0.749 | -0.699 | 1.624 |
| Sample 2 | 23.726 | 23.513 | 0.213 | 0.262 | 0.833 |
| Sample 3 | 23.088 | 22.928 | 0.160 | 0.209 | 0.865 |
| Sample 3 | 23.622 | 23.170 | 0.452 | 0.501 | 0.706 |
| Sample 3 | 22.564 | 22.707 | -0.143 | -0.093 | 1.067 |
| $\bar{\boldsymbol{x}} \boldsymbol{\pm SD}$ = 1,023 ± 0,25 | | | | | |
| Cell line | **Ct (*LIMD2*)** | **Ct (*ACTB*)** | **ΔCt** | **ΔΔCt** | $\boldsymbol{2}^{\boldsymbol{-}\boldsymbol{\Delta\Delta Ct}}$ |
| BCPAP | 29.866 | 29.258 | 0.608 | 0.657 | 0.634 |
| BCPAP | 29.293 | 29.714 | -0.421 | -0.372 | 1.294 |
| BCPAP | 29.396 | 29.403 | -0.007 | 0.042 | 0.971 |
| $\bar{\boldsymbol{x}}\boldsymbol{\pm SD}$ = 0,966 ± 0,33 | | | | | |
| TPC1 | 29.568 | 29.993 | -0.425 | -0.376 | 1.297 |
| TPC1 | 29.388 | 30.105 | -0.717 | -0.668 | 1.588 |
| TPC1 | 28.410 | 29.064 | -0.654 | -0.605 | 1.520 |
| $\bar{\boldsymbol{x}}\boldsymbol{\pm SD}$ = 1,468 ± 0,15 | | | | | |

Results of copy number variation by qPCR showing the mean ($\bar{\mathbf{x}}\mathbf{)}$and standard deviation (ST) of Ct . The relative copy number state was calculated using 2(-ΔΔCt). Copy numbers above 1.5 is defined as gained.
