# Supplementary material for: LIMD2 Regulates Key Steps of Metastasis Cascade in Papillary Thyroid Cancer Cells via MAPK Crosstalk
Source: Cells. 2020 Nov 23;9(11):2522. doi: 10.3390/cells9112522 (PMC7700534; doi:10.3390/cells9112522)
Supplement: Supplementary file 1 [file cells-09-02522-s001.zip › supplementary final/4. Legens of Suplementary Figures.docx]

**Legends of Supplementary Figures**

**Figure S1**. Analysis of off-target results, showing the alignment of nucleotide sequences of parental and edited cells, being observed no divergence among these sequences and the absence of off-target effects.

**Figure S2**. Amino acid sequence alignments of seven different clones isolated from cells transfected with gRNA2. A frameshift mutation from the residue 43, which comprises the second cysteine that binds to a zinc ion within the first zinc-finger domain, promoted the loss of LIM domain and, therefore, adapter property of LIMD2 (highlighted in purple).
