# Supplementary figures and images for: LIMD2 Regulates Key Steps of Metastasis Cascade in Papillary Thyroid Cancer Cells via MAPK Crosstalk
Source: Cells. 2020 Nov 23;9(11):2522. doi: 10.3390/cells9112522 (PMC7700534; doi:10.3390/cells9112522)

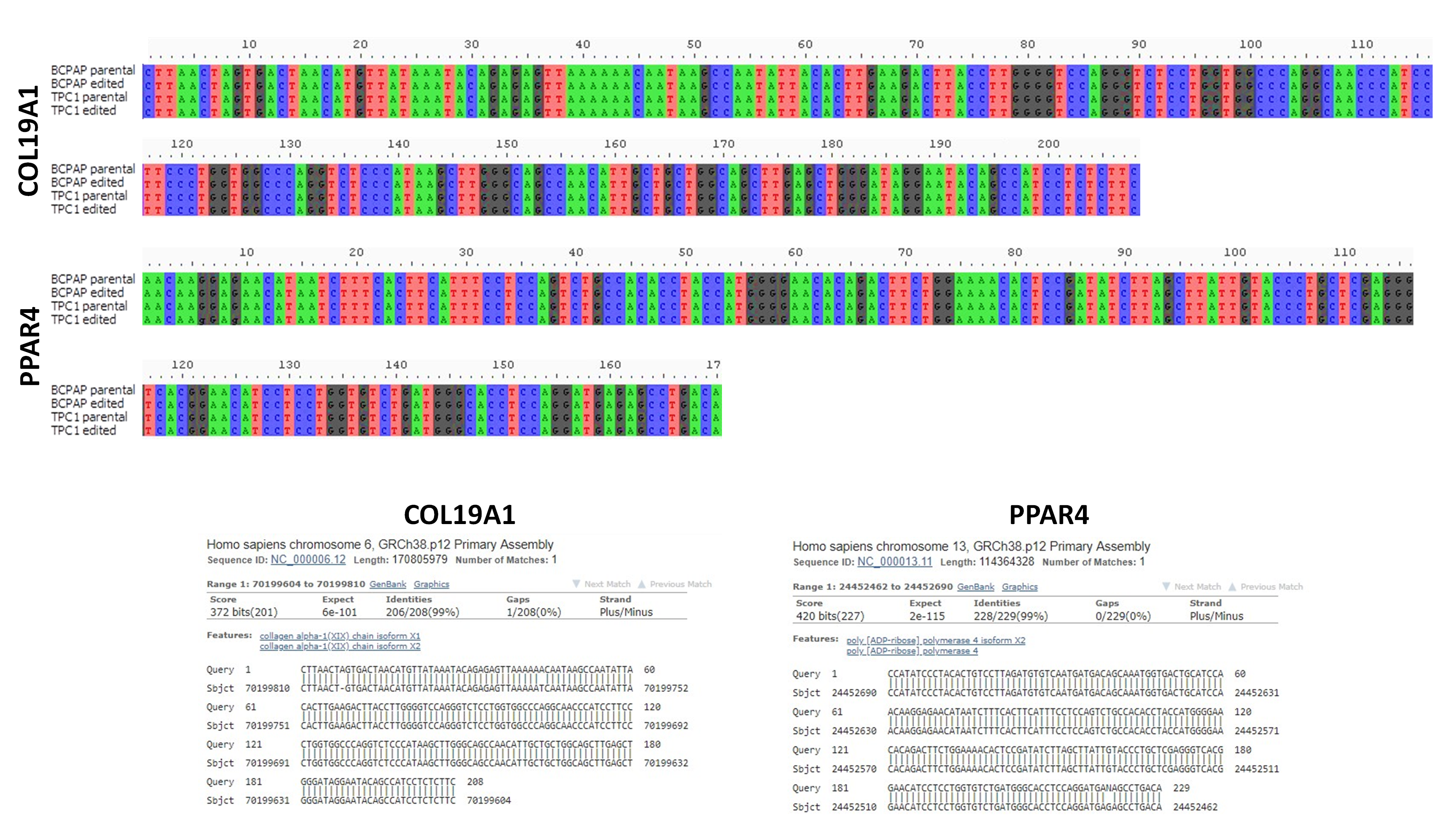

Supplement: Supplementary file 1 [file cells-09-02522-s001.zip › supplementary final/2. Supplementary Figure 1.tif]

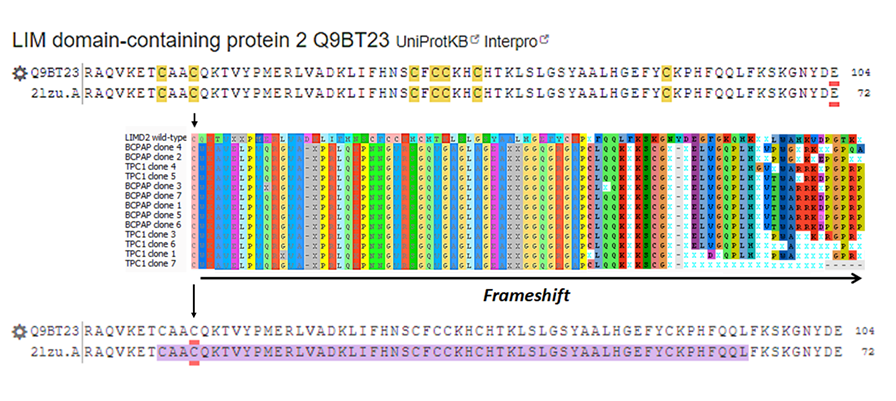

Supplement: Supplementary file 1 [file cells-09-02522-s001.zip › supplementary final/3. Supplementary Figure 2.tif]
